# Sanitary Condition of Louisville

**Published:** 1855-09

**Authors:** 


### SANITARY CONDITION OF LOUISVILLE.

The only diseases which have prevailed to any extent in Louisville during the summer are hooping cough and intermittent fever. The city has been almost wholly exempt from cholera, and not a case has come within our knowledge for weeks past. Bilious fever has presented itself in a few localities, principally in the suburbs of the city, where intermittent fever is prevalent, but the health of the city, at this time, may be pronounced excellent.

